# Intrahepatic interleukin 10 expression modulates fibrinogenesis during chronic HCV infection

**DOI:** 10.1371/journal.pone.0241199

**Published:** 2020-10-30

**Authors:** Ednelza da Silva Graça Amoras, Samara Tatielle Monteiro Gomes, Maria Alice Freitas Queiroz, Mauro Sergio Moura de Araújo, Marialva Tereza Ferreira de Araújo, Simone Regina Souza da Silva Conde, Ricardo Ishak, Antonio Carlos Rosário Vallinoto

**Affiliations:** 1 Virology Laboratory, Biological Science Institute, Federal University of Pará, Belém, Pará, Brazil; 2 School of Medicine, Health Science Institute, Universidade Federal do Pará, Belém, Pará, Brazil; 3 Hepatology Outpatient Clinic, João Barros Barreto University Hospital, Belém, Pará, Brazil; National Institutes of Health, UNITED STATES

## Abstract

**Introduction:**

Liver fibrosis is a result of continuous damage to the liver combined with accumulation of the extracellular matrix and is characteristic of most chronic liver diseases such as hepatitis C virus (HCV) infection.

**Methods:**

This study evaluated interleukin 10 (IL10) expression in the liver and plasma of 45 HCV patients and its association with the pathogenesis and progression of liver fibrosis. The expression of transforming growth factor beta (*TGFB1*) was also assessed. Patients were divided into three groups according to the METAVIR classification (F0-F1, F2 and F3-F4); there was also a control group (n = 8).

**Results:**

In the control group, high intrahepatic *IL10* mRNA expression showed a positive association with F0-F1 fibrosis, no inflammation, low concentrations of liver enzymes and a high viral load; conversely, low intrahepatic *IL10* mRNA expression showed a negative association with fibrosis progression. Intrahepatic *TGFB1* mRNA expression was greater in the HCV group than in the control group, and regarding different disease phases, its expression increased as fibrosis evolved to more severe forms.

**Conclusion:**

Intrahepatic *IL10* mRNA expression decreases with persistent fibrosis, probably due to the production of TGF-β1, a potent antimitotic and fibrogenic cytokine. *IL10* restricts and decreases the immune response and limits the fibrogenic response; however, a decrease in *IL10* favors persistent inflammatory infiltrate, resulting in severe fibrosis.

## 1. Introduction

Liver fibrosis is a result of chronic damage to the liver combined with extracellular matrix accumulation and is characteristic of most types of chronic liver disease. Regardless of the initial cause, continued liver injury causes inflammatory damage, matrix deposition, parenchymal cell death and angiogenesis, leading to progressive fibrosis. The healing matrix accumulates very slowly; however, once cirrhosis is established, the potential to reverse this process is decreased, and complications develop [1]. The liver is a highly immunotolerant organ with high physiological relevance, therefore, the deregulation of the effector and suppressor immune response can induce the persistence of HCV [2]. The cytokines IL-10 and TGF-β1 contribute to an immunotolerant state of the liver, as they inhibit the differentiation of dendritic cells and promote the conversion of naïve CD4 + T cells into regulatory T cells [2, 3]. TGF-β1 is the main cytokine mediator of tissue repair, inducing myofibroblast differentiation, formation of the extracellular matrix, proliferation of fibroblasts and collagen synthesis [4].

IL-10 is an important immunoregulatory and anti-inflammatory cytokine that is produced after antigenic stimulation by several cell populations, including Th2 and Th0 lymphocytes, B cells, dendritic cells and monocytes [5]. IL-10 activity is mediated by its specific cell surface receptor *IL10R*, which is expressed on a variety of cells, especially immune cells [6]. Studies have shown that IL-10 inhibits the proliferation of both Th1 and Th2 cells via cellular anergy [7]. Due to its immunosuppressive activity, IL-10 reduces the effective immune response against various pathogens, favoring its persistence in the body [8]. In the liver, IL-10 production has been documented in hepatocytes, sinusoidal endothelial cells, Kupffer cells, hepatic stellate cells and resident lymphocytes, with prevalent production in the normal liver [9].

IL-10 is related to changes in the inflammatory immune response in viral hepatitis, autoimmune hepatitis, in alcoholic liver disease and its effects have been demonstrated through experimental models [10]. There is evidence that patients with a strong Th1 response during acute HCV infection can eliminate the virus, whereas patients with a Th2 response (elevated IL-10 level) may progress to chronicity [11]. In addition, during treatment after liver transplantation, IL-10 favors immunologic tolerance and may increase allograft survival [11].

The antifibrotic properties of IL-10 were demonstrated in an experimental model of liver cirrhosis [10]. The use of IL-10 as a therapy in patients with HCV induced a reduction in the levels of inflammation, fibrosis score and decreased the activation of Th1 cells, showing its effect in controlling the inflammatory and fibrotic process. However, long-term administration of IL-10 favors an increase in HCV viral load due to changes in the immune response effective in eliminating the virus [12].

The objective of the present study was to quantify *IL10* and *TGFB1* gene expression in liver biopsy specimens from patients with HCV chronic hepatitis and to determine its roles in the pathogenesis and clinical presentation of this infection as well as in the various stages of fibrosis and liver inflammation according to the French METAVIR classification.

## 2. Materials and methods

### Study population

The study group consisted of 53 subjects who were consecutive cases of untreated chronic carriers of HCV (n = 45) treated at the Hepatology Outpatient Clinic of the Hospital of the Fundação Santa Casa de Misericórdia do Pará (FSCMPA) in the period from 2011 to 2015. A control group (n = 8) composed of individuals who underwent conventional cholecystectomy without hepatic necroinflammatory changes and were treated at the Surgery Unit of the João de Barros Barreto University Hospital/UFPA was also included in this study. The patients were divided into the following three groups according to the liver disease stage as defined by the METAVIR classification [13]: without fibrosis and/or mild fibrosis (F0-F1), moderate fibrosis (F2) and severe fibrosis and/or cirrhosis (F3-F4). Normal liver samples were used as a control (CT). Further details on the patient selection criteria can be found in our previous report [14]. Patients with hepatocarcinoma (HCC), those coinfected with HIV and those being treated for hepatitis B and C were excluded from the study.

### Ethical aspects

The present study was submitted to and approved by the Research Ethics Committee of Fundação Santa Casa de Misericórdia do Pará (protocol numbers 117/2009 and 684.432/2014) following the Directives and Standards Regulating Human Research (Resolution 196 of the Brazilian National Health Council). All individuals who agreed to participate in the study signed an informed consent form.

### Sample collection

Liver biopsy specimens were obtained from patients with medical indications to investigate changes in the liver parenchyma. The biopsies were performed with a Tru-Cut needle and guided by ultrasound. Each sample was separated into two sections. The first section was used for a histopathological examination after hematoxylin and eosin (HE), chromotrope aniline blue (CAB), Gomori reticulin and Shikata orcein staining at the UFPA Department of Pathological Anatomy. The diagnosis was based on the French METAVIR classification; portal and periportal inflammatory activity was scored from 0 to 3, and structural changes were scored from 0 to 4. The results of the histopathological exams were obtained from the patients' medical records, which were accessed from 2011 to 2015.

The second section of the biopsy was sent for genetic analysis at the Virology Laboratory/Biological Science Institute-ICB/UFPA and stored at -70°C prior to use. Additionally, blood samples were collected in vacuum tubes containing EDTA as an anticoagulant, and plasma was separated by centrifugation and stored at -20°C prior to measurement of the alanine aminotransferase (ALT), aspartate aminotransferase (AST), gamma-glutamyl transferase (GGT) and viral marker levels.

### RNA extraction and reverse transcription

The liver tissue fragment was stored in 500 μL of RNAlater® Tissue Collection Solution for preservation of the RNA, which was subsequently extracted using a Norgen Biotek Corporation Kit according to the manufacturer’s protocol. The RNA samples were transcribed into complementary DNA (cDNA) using a High-Capacity cDNA Reverse Transcription Kit (without inhibitor) (Applied Biosystems, USA) as previously described [13].

### Quantitative real-time PCR (qPCR)

qPCR was performed in 96-well plates using TaqMan^™^ (Applied Biosystems, USA) on a StepOnePlus system (Life Technologies, Carlsbad, CA, USA). For both the patient and control groups, *IL10* (Hs00174086_m1), *TGFB1* (Hs00171257_m1) and glyceraldehyde-3-phosphate dehydrogenase (*GAPDH*) (Hs02758991_g1) gene expression assays (P/N 4326317E, Life Technologies, CA, USA) were performed in separate wells (singleplex assays). The primers were purchased from Life Technologies (Carlsbad, CA, USA). The relative expression of each gene was determined as previously described [13].

### Measurement of the plasma IL-10 levels

The plasma IL-10 levels were measured using a Bio-Plex ProTM Human Cytokine 27-Plex Kit (Bio-Rad, CA, USA) and read on a LUMINEX 200 system following the manufacturer's protocols.

### Dosage of liver enzymes

The measurement of ALT, AST and GGT enzymes was carried out by colorimetric enzymatic reaction with automated methodology, using the Architect-Abbott c800 equipment (Chicago, Illinois, USA), following instructions provided by the manufacturer.

### Plasma viral load

Plasma viral load was measured by real-time PCR using an Abbott RealTime HCV Amplification Reagent Kit (Abbott Park, Illinois, USA) at the Central Laboratory of the State of Pará (LACEN-PA). HCV viral load quantifications are presented as copies/mL and log10 value. The lowest detection level of HCV load was 1.08 log UI/mL, and the highest detection level was 8.00 log UI/mL.

### Statistical procedures

Differences between groups were analyzed using the Kruskal-Wallis and Mann-Whitney U tests. Relationships among the variables were determined by Spearman's correlation analysis. The level of significance was set at 5% (p value ≤ 0.05) using BioEstat 5.0 and GraphPad Prism version 6.1 software. For significant data, heatmap matrices were plotted using R 3.4.2 software with canberra and ward.D as the distance and cluster methods, respectively, depending on the options provided by the software.

Biomarker networks were obtained to evaluate correlations among the biomarkers and were analyzed using Cytoscape software. The networks were constructed using edges, representing the Spearman correlation scores, which were characterized as strongly positive (r ≥ 0.68, dark solid line), moderately positive (0.36 ≤ r ≤ 0.67; light solid line) and negative (-0.37 ≥ r; dashed line).

## 3. Results

In the present study, the mean patient age was 48.3 years, with 21 (46.7%) patients being female and 24 (53.3%) being male. After separating the patients according to their liver disease stage, the group of patients without fibrosis or with mild fibrosis (F0-F1) comprised the largest number of individuals, followed by those with severe fibrosis and/or cirrhosis (F3-F4) and intermediate fibrosis (F2). The median ALT, AST and GGT concentrations were elevated in all stages of the disease and were within normal values only in the control group. Inflammation (A2) and ALT levels were highest in the group with intermediate fibrosis (F2), and AST and GGT levels were highest in the group with severe fibrosis and/or cirrhosis (F3-F4) (Tables 1 and 2).

**Table 1:** Clinical, biochemical, and histopathological information on the study population.

Variables	Chronic hepatitis C	Normal control
Subjects (*n*)	45	8
Gender (F/M) (%)	21/24 (46.7/53.3)	4/4 (50.0/50.0)
Age (mean)	47.8	39.8
ALT (IU/L) Median	79.0	27.5
AST (IU/L) Median	66.3	21.0
GGT (IU/L) Median	65.9	29.0
Fibrosis Scores^a^		
F0 (%)	6 (13.3)	8 (100.0)
F1 (%)	20 (44.4)	-
F2 (%)	8 (17.8)	-
F3 (%)	5 (11.2)	-
F4 (%)	6 (13.3)	-
Level of Inflammation^b^		
A0 (%)	6 (13.3)	8 (100.0)
A1 (%)	24 (53.3)	-
A2 (%)	15 (33.4)	-

ALT: Alanine aminotransferase (ref.: 14 to 55 IU/L); AST: Aspartate aminotransferase (ref.: 14 to 32 IU/L); GGT: Gamma-glutamyl transferase (ref.: < 50 IU/L)

^a^Fibrosis scores: F0, absence of fibrosis; F1, portal fibrosis without septa; F2, portal fibrosis with rare septa; F3, numerous septa without cirrhosis; and F4, cirrhosis.

^b^Inflammatory activity: A0, absent; A1, minimum; and A2, moderate.

**Table 2:** Clinical, biochemical and histopathological data according to hepatic changes (METAVIR) in the study population.

Variables	Liver fibrosis score—METAVIR^a^			
	F0-F1	F2	F3-F4	CT
	(n = 26)	(n = 8)	(n = 11)	(n = 8)
Liver enzymes				
ALT (IU/L) Median	67.0	122.3	85.3	27.0
AST (IU/L) Median	53.2	77.5	83.2	21.0
GGT (IU/L) Median	50.0	92.0	128.0	29.0
Inflammatory activity^b^ n (%)				
A0	6 (26.00)	0	0	8 (100)
A1	16 (61.60)	2 (25.00)	6 (54.50)	0
A2	4 (15.40)	6 (75.00)	5 (45.50)	0

ALT: Alanine aminotransferase (ref.: 14 to 55 IU/L); AST: Aspartate aminotransferase (ref.: 14 to 32 IU/L); GGT: Gamma-glutamyl transferase (ref.: < 50 IU/L)

^a^Fibrosis scores: F0, absence of fibrosis; F1, portal fibrosis without septa; F2, portal fibrosis with rare septa; F3, numerous septa without cirrhosis; and F4, cirrhosis.

^b^Inflammatory activity: A0, absent; A1, minimum; and A2, moderate.

**Fig 1:**
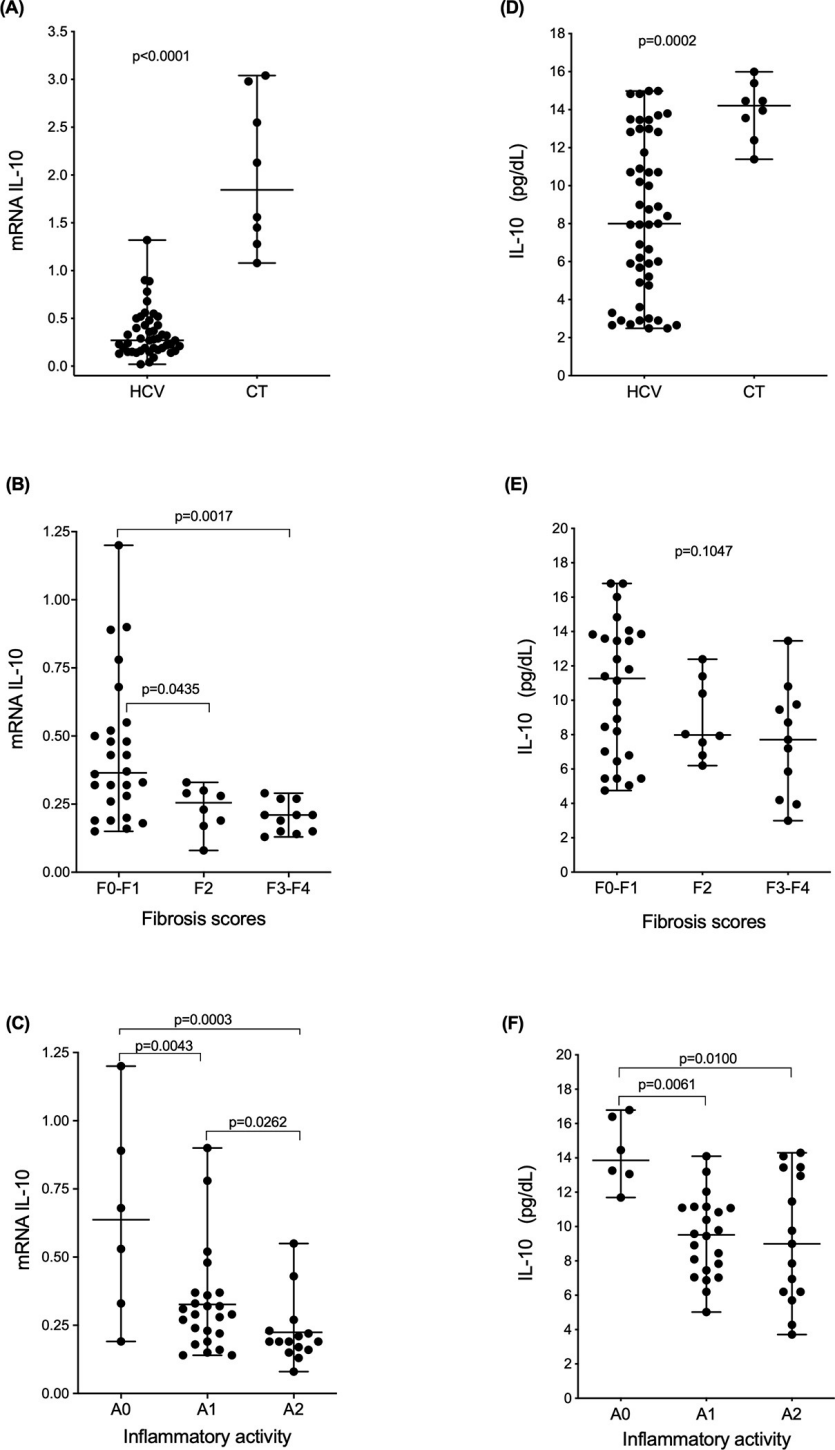
*IL10* mRNA and plasma IL-10 levels according to the clinical condition of the liver. A-C: Intrahepatic *IL10* expression between the HCV and control (CT) patients with different fibrosis scores and with different necroinflammatory activity levels (A: Mann-Whitney test; B and C: Kruskal-Wallis test). D-F: Plasma IL-10 concentrations between HCV and CT patients with different fibrosis scores and be with different necroinflammatory activity scores (D: Mann-Whitney test; E and F: Kruskal-Wallis test).

Intrahepatic and plasma IL-10 levels were significantly higher in the control group (CT) (median mRNA: 1.84; IQR: 1.55 and median plasma concentration: 14.21; IQR: 2.480) than in the group with chronic hepatitis C (median mRNA: 0.24; IQR: 0.300 and median plasma concentration: 7.95; IQR: 10.050) (p<0.0001 and p = 0.0004, respectively; Fig 1A and 1D). Regarding the disease stage, the cytokine expression levels were highest in patients with fibrosis scores F0-F1 (median: 0.365; IQR: 0.285) and decreased significantly (p = 0.0435 and p = 0.0002) as liver disease progressed to moderate fibrosis (F2) (median: 0.255; IQR: 0.1100) and severe fibrosis and cirrhosis (F3-F4) (median: 0.210; IQR: 0.123) (p<0.0001 compared to the control group; Fig 1B). The same trend was observed for inflammation, in which the patients without liver inflammation (A0) had significantly higher *IL10* expression levels (median: 0.6050; IQR: 0.688) than the patients with mild (A1) (median: 0.2900; IQR: 0.170) and moderate (A2) (median: 0.1900; IQR: 0.070) inflammatory activity (A0 compared to A1 and A2, p = 0.0043 and p = 0.0003, respectively; and A1 compared to A2 and the control, p = 0.0262 and p = 0.0013, respectively; Fig 1C). The association between the serum IL-10 levels and METAVIR scores were the same [F0-F1 (median: 11.27; IQR: 7.666), F2 (median: 7.980; IQR: 4.151) and F3-F4 (median: 7.710; IQR: 5.560)], but the differences were not significant; however, when the F0-F1 group was compared to the control group, the difference was significant (p = 0.0269; Fig 1E). Concerning inflammatory activity, the plasma concentrations were significantly higher in the group without inflammation (A0) (median: 13.86; IQR: 3.77) than in the groups with inflammation [A1 (median 9.516; IQR: 3.76) and A2 (median: 8.995; IQR: 7.252)](p = 0.0061 and p = 0.0100, respectively). However, the difference between the group without inflammation (A0) and the control group was not significantly different (p = 0.9754; Fig 1F).

**Fig 2:**
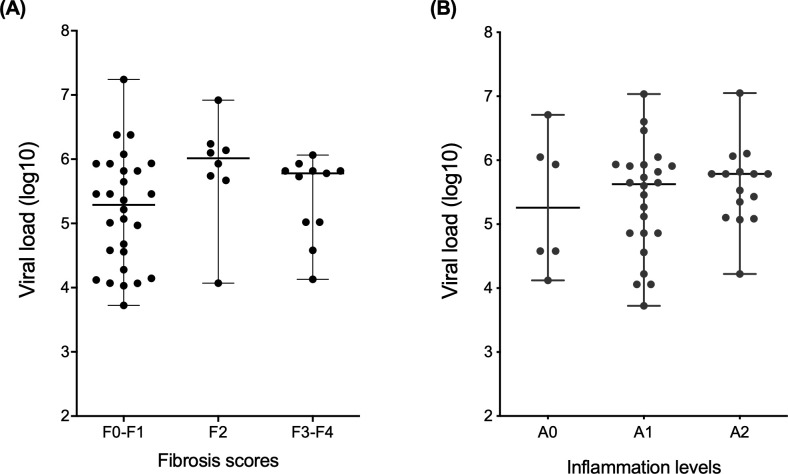
HCV viral load according to the clinical condition of the liver. A-B: Comparison of the median plasma viral load in chronic viral hepatitis patients with different fibrosis scores and with necroinflammatory activity scores (METAVIR) in the liver. Kruskal-Wallis test.

When patients were grouped according to viral load (log10) (i.e., fibrosis score), the highest median was observed in patients in the intermediate stage of liver disease (F2) (median: 6.015; IQR: 0.527), followed by those in stages F3-F4 (median: 5.780; IQR: 0.797) and stages F0-F1 (median: 5.290; IQR: 1.551), although the difference was not statistically significant (Fig 2A). When grouped according to necroinflammatory activity, the mean plasma viral load was higher in patients in group A2 (median: 5.785; IQR: 0.715), followed by those in groups A1 (median: 5.625; IQR: 1.064) and A0 (median: 5.357; IQR: 1.749), although the difference was not statistically significant (Fig 2B).

**Fig 3:**
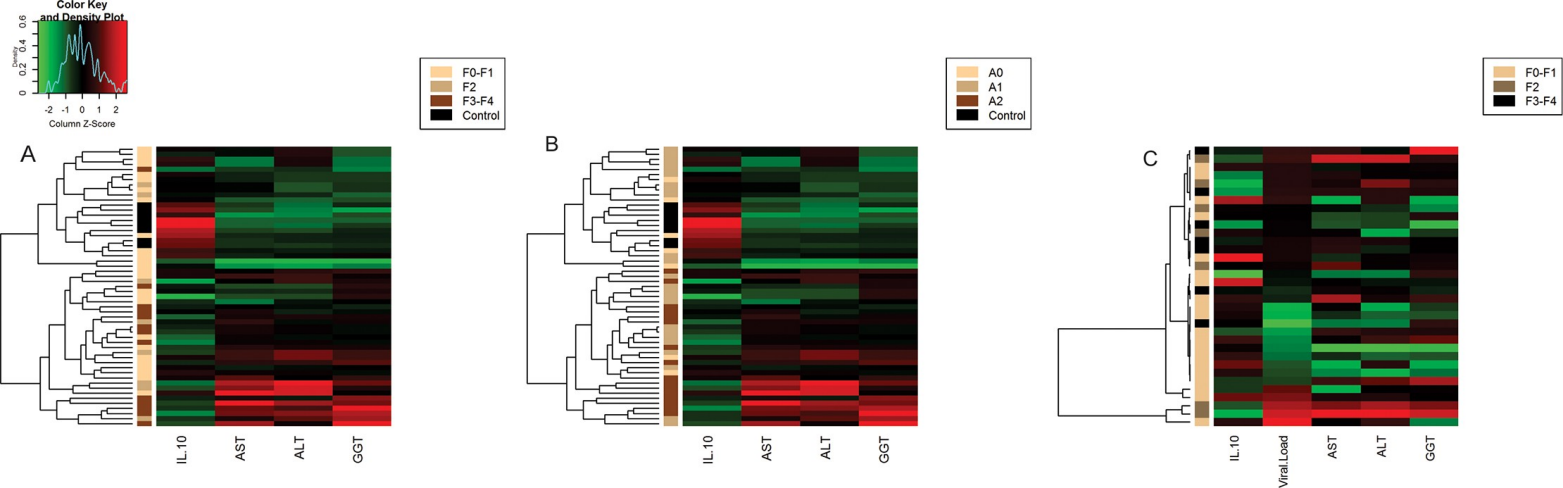
Analysis of *IL10* mRNA levels according to the clinical condition of the liver and viral load represented by heatmaps. A-B: Intrahepatic *IL10* expression showed a clear ability to cluster patients with low plasma ALT, AST and GGT concentrations and different METAVIR scores. C: Viral load was able to cluster patients with high plasma concentrations of liver enzymes, but intrahepatic *IL10* expression was not able to cluster chronic liver disease patients with different liver fibrosis scores (METAVIR).

The heatmap (Fig 3A) illustrates the ability of *IL10* expression to group patients according to liver disease progression and the plasma concentrations of liver enzymes. Fig 3A clearly shows increased expression patterns of this cytokine clustered in individuals without hepatic disease (CT), those with low fibrosis scores (F0-F1) and those with low plasma ALT, AST, GGT concentrations. Conversely, low *IL10* expression was able to group patients with the highest fibrosis scores and the highest concentrations of the enzyme markers. Fig 3B shows the same clustering trend of *IL10* expression relative to the liver inflammation levels and biochemical markers. Fig 3C shows that viral replication was not directly associated with intrahepatic *IL10* expression in the chronic stages of liver disease; however, we observed small clusters grouping patients with increased viral loads in the intermediate and final stages of liver disease, although the clustering ability of *IL10* expression relative to the viral load was not evident.

**Fig 4:**
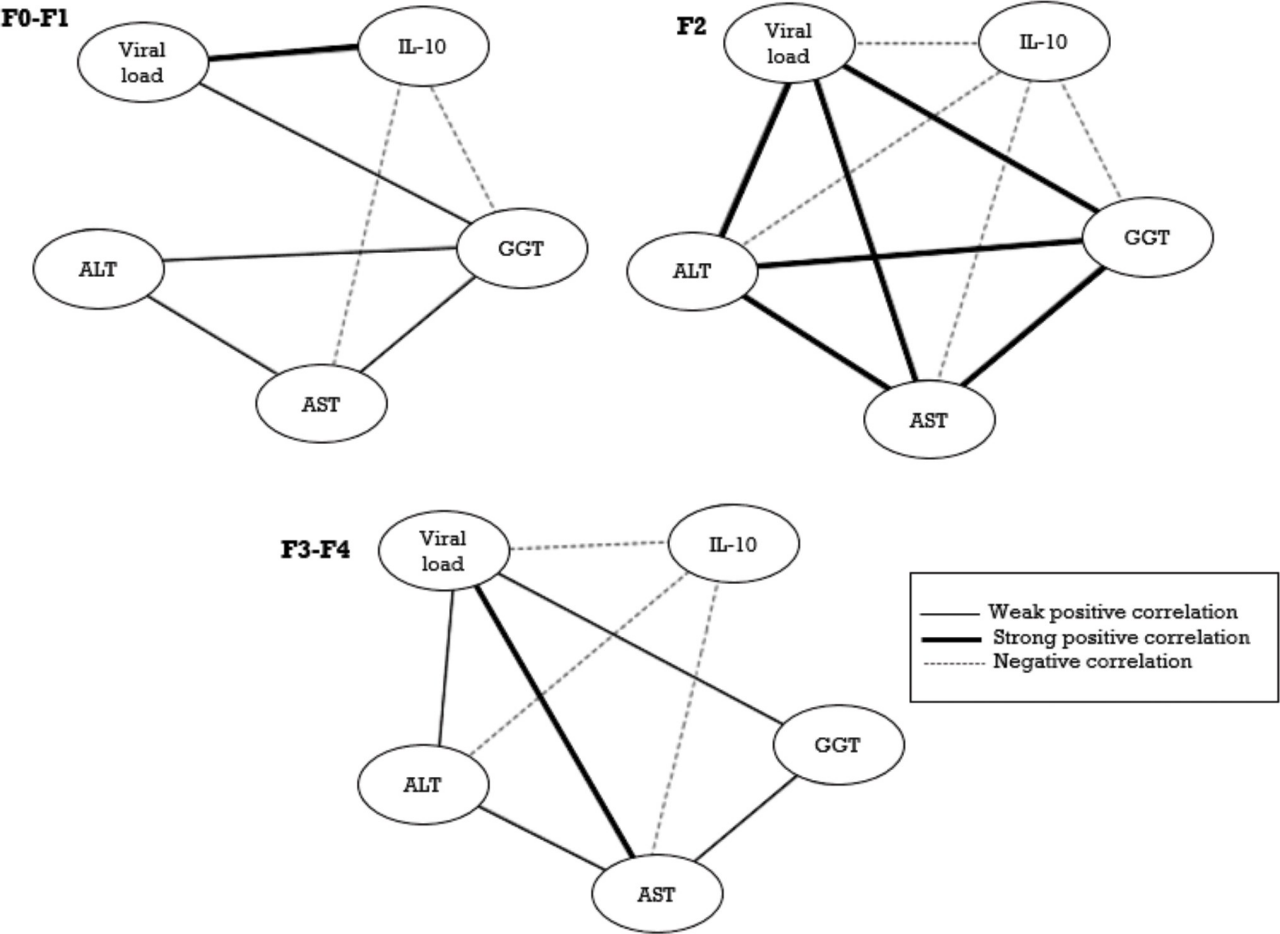
Interaction networks between *IL10* mRNA levels and the analyzed biomarkers. Interaction networks between the analyzed biomarkers. Correlation values were categorized and are represented by connecting lines (edges). The r values were used to categorize strong positive correlations (r ≥ 0.68, dark solid), moderate positive correlations (0.36 ≤ r ≤ 0.67; light solid line) and negative correlations (-0.37 ≥ r; dashed line). Spearman's correlation analysis was used.

An interaction network between the study variables was constructed through Spearman’s correlations. We observed a negative association between *IL10* expression and plasma ALT, AST and GGT concentrations at all stages of chronic liver disease. However, we also observed an increase in liver enzymes in the intermediary stage of liver fibrosis (F2) that was strongly associated with viral load. This association was weak in the initial stages, whereas in the final stages, only AST showed an association with the viral load levels. In the F0-F1 stages, a strong positive association was found between increased intrahepatic *IL10* mRNA expression and an increased viral load. However, in the intermediate stage (F2) and in the late stages (F3-F4), a negative association was found between an increased viral load and decreased intrahepatic *IL10* mRNA expression (Fig 4).

**Fig 5:**
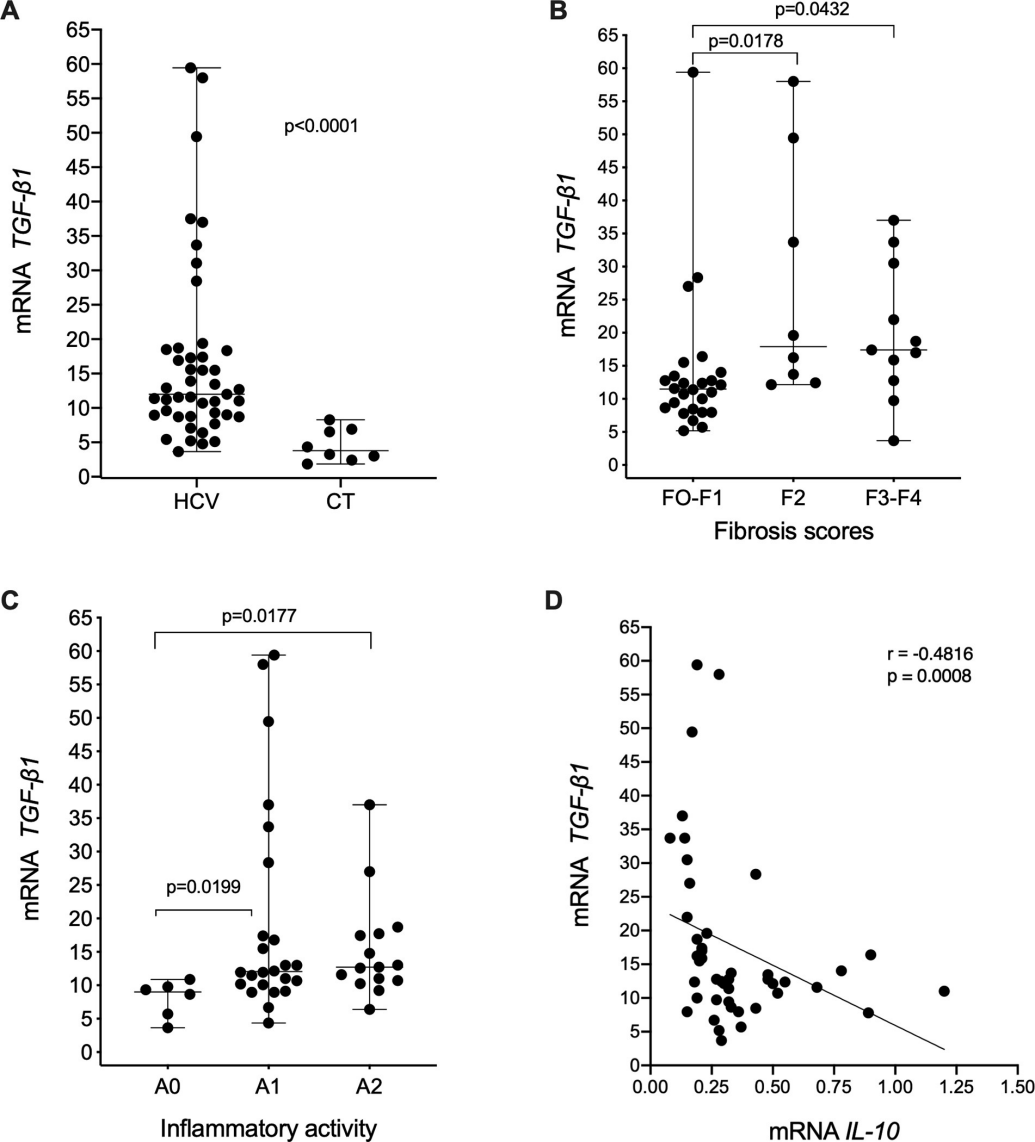
*TGFβ1* mRNA level according to the clinical condition of the liver and its correlation with the *IL10* mRNA level. A-C: Intrahepatic *TGFβ1* expression between HCV and CT patients (Mann-Whitney test) and between patients with different fibrosis scores and with different necroinflammatory activity levels (Kruskal-Wallis test). D: Spearman's correlation analysis between *TGFB1* and *IL10* mRNA levels.

Fig 5 shows significantly higher *TGFB1* expression in the HCV group (median mRNA: 12.901; IQR: 9.070) than in the CT group (median mRNA: 3.791; IQR: 4.237; p<0.0001; Fig 5A). According to the disease stage, the lowest levels of *TGFB1* expression were observed in patients with a fibrosis score of F0-F1 (median mRNA: 11.410; IQR: 5.255), with a significant increase in gene expression (p = 0.0178 and p = 0.0432) as the liver disease evolved to F2 (median mRNA: 17.910; IQR: 32.75) and F3-F4 (median mRNA: 17.390; IQR: 17.730; Fig 5C). The same trend was observed with respect to necroinflammatory activity, in which *TGFB1* expression was significantly lower in patients without liver inflammation (A0) (median mRNA: 8.990; IQR: 4.860) than in those with mild (A1) (median mRNA: 12.05; IQR: 15.520) and moderate (A2) (median mRNA: 12.712; IQR: 7.031) necroinflammatory activity (p = 0.0199 and p = 0.0177, respectively; Fig 5B). Fig 5D shows a significant negative correlation (r = -0.4816 and p = 0.0008) between intrahepatic *IL10* and *TGFB1* mRNA levels.

## 4. Discussion

In the present study, we observed higher intrahepatic *IL10* mRNA expression levels in the control group than in the group with chronic hepatitis. IL-10 is produced by Th2 cells, and patients with chronic HCV infection show a predominance of a Th2 response that hinders Th1-dependent viral clearance. A concomitant increase in the action of Treg cells (CD25^+^) is believed to increase IL-10 production, thereby suppressing the Th1 response stimulated by viral proteins [15]. In addition, physiologically, the liver has a tolerogenic environment, and this suppressive condition occurs as a result of the constitutive exposure of hepatic cells to traces of endotoxins and other bacterial lipopolysaccharide (LPS) products, among other reasons, which results in the low modulation of costimulatory molecules and IL-10 synthesis by Kupffer and sinusoidal endothelial cells [16]. We also observed the same trend in the plasma IL-10 concentrations in the control and patient groups, confirming previous results [17].

HCV patients without fibrosis and without necroinflammation had lower plasma and intrahepatic levels of IL-10 protein and mRNA than controls, suggesting that this profile may be related to the absence of viral clearance, which is characteristic of chronic infection by HCV [15, 16].

High intrahepatic *IL10* mRNA expression in the F0-F1 fibrosis and A0 and A1 inflammatory activity stages indicates an association of this cytokine with viral persistence and an immunosuppressive role in the early stages of chronic hepatitis. Specifically, the plasma IL-10 concentration was also increased, but the difference was not significant. This immunosuppressive mechanism of IL-10 in the liver occurs when soluble antigens that pass through the liver sinusoids are absorbed by sinusoidal endothelial cells and are presented to both CD8^+^ and CD4^+^ T cells, which are induced to proliferate but are unable to maintain IL-2 and IFN-γ secretion [18]. CD8^+^ T cells are unable to differentiate into effector cytotoxic cells, whereas CD4^+^ T cells can differentiate into an anti-inflammatory IL-4 and IL-10 secretory phenotype [18]. The low levels of IL-10 (both plasma and mRNA) observed in the groups with fibrosis (F2 and F3-F4) and with high inflammation (A2) strongly suggest that IL-10 helps control the formation of inflammation-induced fibrosis, consistent with a previous report [17].

Additionally, previous studies have reported a protective role for IL-10 in HCV infection in relation to the progression of liver fibrosis [19]. Other studies have emphasized the protective role of IL-10 during the treatment of chronic hepatitis C infection: it reduced the severity of fibrosis in participants [12]. In other studies with animal models, the absence of IL-10 was associated with liver fibrosis [20, 21].

Intrahepatic *IL10* mRNA expression seems to reflect the blood pattern of this cytokine, corroborating a previous report in which HCV patients with severe fibrosis (F3-F4) had a lower frequency of intrahepatic T cells (especially Tregs) than patients with mild hepatic fibrosis. In the same study, patients with mild liver fibrosis had higher serum levels of IL-10 than patients with severe liver disease (F3-4), demonstrating similar serum and tissue levels of cytokines in HCV patients and supporting the use of serum cytokines as biomarkers associated with the liver fibrosis score [22].

Our results showed that regarding established liver fibrosis, a score of F2 was associated with the highest levels of viral replication, followed by F3-F4. High levels of inflammation (A2) in F2 corroborate data from the literature that after immune tolerance, immune activation occurs, with increased viral replication, causing inflammation and hepatocyte necrosis, which can be reflected by the increase in liver enzymes together with the regeneration of liver cells and marked fibrous cell hyperplasia [16].

Intrahepatic *IL10* mRNA expression showed a positive association with an increased plasma viral load in the early disease stages (F0-F1); however, this association was not observed in the intermediate (F2) and late (F3-F4) stages. In the F2 and F3-F4 stages, a positive correlation between viral persistence and the hepatic necroinflammation markers ALT, AST and GGT was observed, with the highest significance found in the intermediate stage of fibrosis (F2), at which point the highest levels of necroinflammatory activity (A2) were also observed. In this context, our results also showed an increase in the serum IL-10 levels in the early stages of fibrosis (F0-F1) and a decline in the final stages, with statistically significant differences. Some studies have shown that elevated IL-10 levels correlate with decreased T cell activity in patients with HCV and the inability of these cells to control viral replication, which emphasizes the important role of T cell depletion in potentiating viral persistence. This finding suggests that the outcome of the immune response for the prevention of viral persistence is not dictated by the initially high viral replication levels; in contrast, a generalized infection can be quickly contained by the maintenance of T cell activity [23]. A study of a cohort of women with persistent HCV infection revealed that CD4^+^ T cells secreted IFN-γ and IL-10 in response to the core protein and demonstrated that CD4^+^ and Treg T cells were induced against the same epitopes of the core protein during HCV infection [24].

Experimental studies in cell cultures have shown that HCV induces an increase in IL-10 in CD4^+^ T in lymphocyte cell lines [25]. The presence of IL-10 in the culture of naive CD8^+^ T cells reduced the frequency of specific cells, showing an association between the early production of IL-10 and the failure to activate specific T cells and viral persistence [26].

Our results showing a negative association between intrahepatic *IL10* mRNA expression and increased ALT, AST and GGT levels with the progression of liver disease to cirrhosis corroborate reports in the literature demonstrating the antifibrotic and antifibrogenic roles of IL-10 in liver damage. This finding is in agreement with results showing increased intrahepatic *IL10* mRNA expression levels in the early stage of fibrosis that disappear in advanced stages, suggesting that IL-10 released by hepatic stellate cells (HSCs) suppresses collagen production through a negative self-regulatory role following the induction of collagenase during the early stage of liver fibrosis. On the other hand, the lack of intrahepatic *IL10* mRNA expression by HSCs in association with the induction of collagenase leads to advanced liver fibrosis [24]. Furthermore, in HCV infection, specific *IL10* polymorphisms are correlated with increased susceptibility to the development of chronic infection and increased severity of hepatic disease [27].

TGF-β1 participates in different stages of liver disease progression and in the activation and differentiation of HSCs, with subsequent stimulation of extracellular matrix proteins, such as collagen and fibronectin, inducing fibrosis [1, 28]. In our study, the intrahepatic expression of *TGFB1* was approximately four times higher in HCV carriers than in controls; moreover, a significant increase in this gene was associated with the highest levels of necroinflammatory activity and high liver fibrosis scores, demonstrating that in chronic hepatitis, with the failure of the immune system to control viral replication, there is a persistent recruitment of mononuclear inflammatory infiltrate, leading to chronic inflammation with sustained liver damage that is modulated by several factors, including TGF-β1 and IL10 [29, 30]. Furthermore, the negative correlation between the highest levels of *TGFB1* mRNA expression levels and low intrahepatic *IL10* mRNA expression levels in the livers of individuals with HCV demonstrates that chronically infected patients have decreased IL-10 levels with the continuous stimulation of factors that lead to the synthesis of TGF-β1, with the consequent progression of fibrosis and evolution to cirrhosis, corroborating previous studies [30, 31].

Taken together, our results demonstrate that initially, the constitutive expression of *IL10* in the liver induces the tolerance of virus-specific CD8^+^ T cell infiltrates, decreases their proliferative capacity and results in the loss of antiviral effector functions, which are important for the decrease in immunopathogenesis at the expense of viral persistence. In addition, increased intrahepatic *IL10* mRNA expression is likely to maintain HSCs in a quiescent stage by suppressing their profibrogenic function in the early disease stages. However, intrahepatic *IL10* mRNA expression decreases with persistent fibrosis, probably due to the perpetuation of activated HSCs with production of the extracellular matrix and TGF-β1, which is considered a potent antimitotic and fibrogenic cytokine, thereby making liver damage irreversible [32, 33].

## Conclusion

The inhibitory activity of IL-10 produced locally in the liver can limit and weaken the immune response, promote viral persistence and indirectly limit the fibrogenic response by controlling TGF-β1 secretion. However, a decrease in hepatic IL-10 favors an increase in persistent inflammatory infiltrate, resulting in severe fibrosis and cirrhosis.

## Supporting information

S1 Dataset (PDF)
