# Supplementary material for: Intrahepatic interleukin 10 expression modulates fibrinogenesis during chronic HCV infection
Source: PLoS One. 2020 Oct 30;15(10):e0241199. doi: 10.1371/journal.pone.0241199 (PMC7598451; doi:10.1371/journal.pone.0241199)
Supplement: S1 Dataset — (PDF) [file pone.0241199.s001.pdf]

Order	Biopsy date	Gender	Age	Diagnóstic	Inflammatory activity	Fibrosis score	ALT	AST	GGT	mRNA *IL10*	mRNA *TGFB1*	IL-10 (pg/dL)
1	23.09.11	M	39	HCV	A1	F1	28	20	24	0,33	8,65	13,46
2	21.10.11	M	40	HCV	A0	F1	55	57	55	0,30	27,01	16,79
3	04.11.11	M	40	HCV	A1	F1	46	42	47	0,89	7,80	5,45
4	18.11.11	F	44	HCV	A2	F1	33	24	77	0,18	12,37	13,46
5	18.11.11	F	45	HCV	A2	F1	52	15	55	0,19	10,01	13,58
6	25.02.11	F	48	HCV	A0	F0	78	82	70	0,19	16,40	14,36
7	18.03.11	F	32	HCV	A1	F1	69	61	50	0,32	12,37	16,79
8	06.05.11	F	49	HCV	A1	F1	72	56	50	0,55	9,44	4,28
9	27.05.11	M	52	HCV	A2	F1	55	35	65	0,28	5,19	13,86
10	02.06.11	F	54	HCV	A1	F0	31	45	29	0,52	6,70	9,88
11	19.08.11	F	51	HCV	A1	F1	257	255	62	0,43	28,36	3,95
12	01.09.11	M	62	HCV	A0	F1	35	35	26	0,89	15,50	13,46
13	21.09.11	F	36	HCV	A1	F1	79	15	16	0,32	11,39	16,80
14	18.11.11	F	42	HCV	A1	F0	35	35	26	0,68	7,95	8,20
15	25.11.11	F	55	HCV	A1	F1	87	43	21	0,48	13,45	14,83
16	10.02.12	F	45	HCV	A0	F1	87	43	21	0,53	5,70	11,80
17	02.03.12	F	47	HCV	A1	F1	112	89	65	0,48	12,77	12,39
18	13.04.12	F	38	HCV	A1	F1	55	37	58	1,23	11,02	8,45

19	27.04.12	F	52	HCV	A1	F1	145	89	89	0,78	14,02	14,46
20	06.06.12	M	46	HCV	A1	F1	39	34	42	0,43	8,47	13,59
21	08.08.12	F	39	HCV	A0	F1	79	15	16	1,20	12,13	5,45
22	23.08.12	F	35	HCV	A1	F1	57	110	80	0,68	11,58	13,83
23	13.12.12	M	48	HCV	A1	F0	89	45	87	0,26	10,71	6,45
24	10.01.13	M	39	HCV	A2	F1	36	34	72	0,15	7,95	13,46
25	31.01.13	M	49	HCV	A1	F0	100	85	114	0,32	12,77	8,92
26	14.03.13	M	39	HCV	A2	F2	16	10	10	0,19	16,23	9,76
27	18.04.13	M	61	HCV	A2	F2	257	132	75	0,33	13,70	6,80
28	25.04.13	F	46	HCV	A2	F2	38	40	18	0,17	19,59	13,46
29	10.05.13	F	39	HCV	A1	F2	31	45	29	0,28	58,00	8,03
30	08.08.13	M	39	HCV	A2	F2	286	142	155	0,23	12,44	5,05
31	19.09.13	F	48	HCV	A2	F2	145	89	89	0,08	49,45	5,45
32	31.10.13	M	54	HCV	A2	F2	55	88	68	0,29	33,70	7,55
33	08.11.13	M	48	HCV	A1	F2	136	74	239	0,30	12,14	3,79
34	21.11.13	F	47	HCV	A1	F3	40	74	50	0,27	18,71	5,85
35	21.03.14	M	65	HCV	A1	F4	33	24	77	0,29	9,73	9,46
36	04.04.14	M	55	HCV	A2	F3	55	54	40	0,21	3,68	8,71
37	16.04.14	F	53	HCV	A2	F4	129	115	376	0,14	21,98	3,00
38	05.05.14	M	55	HCV	A1	F4	69	61	50	0,19	30,50	10,81
39	23.05.14	M	64	HCV	A2	F4	79	65	70	0,15	16,99	3,95
40	10.06.14	M	53	HCV	A1	F3	75	140	447	0,27	33,70	13,46
41	21.06.14	M	52	HCV	A2	F4	244	207	183	0,21	17,39	4,20
42	04.08.14	M	47	HCV	A1	F3	48	76	237	0,21	37,01	7,71
43	26.08.14	M	50	HCV	A2	F3	36	28	14	0,15	12,77	3,00
44	05.11.14	M	59	HCV	A1	F4	160	117	280	0,13	15,87	9,76

45	10.03.15	M	50	HCV	A0	F0	16	10	10	0,36	59,40	12,39
1	06.03.12	M	39	Normal control	A0	F0	19	24	12	1,45	3,00	14,46
2	06.03.12	F	29	Normal control	A0	F0	35	18	33	1,56	3,26	11,39
3	19.03.12	F	35	Normal control	A0	F0	33	26	29	2,13	1,87	13,56
4	09.05.12	F	62	Normal control	A0	F0	25	19	26	2,55	2,43	15,39
5	21.05.12	F	39	Normal control	A0	F0	29	18	19	3,04	4,32	14,46
6	21.05.12	M	40	Normal control	A0	F0	16	14	22	1,28	8,27	12,39
7	03.06.12	M	32	Normal control	A0	F0	22	27	16	2,98	6,54	13,96
8	03.06.12	M	42	Normal control	A0	F0	22	32	19	1,08	6,90	15,99
